# Improving path planning for mobile robots in complex orchard environments: the continuous bidirectional Quick-RRT* algorithm

**DOI:** 10.3389/fpls.2024.1337638

**Published:** 2024-05-13

**Authors:** Lei Ye, Jin Li, Pu Li

**Affiliations:** School of Intelligent Engineering, Shaoguan University, Shaoguan, China

**Keywords:** path planning, mobile robots, orchard environments, rapidly exploring random tree, bidirectional expansion

## Abstract

Efficient obstacle-avoidance path planning is critical for orchards with numerous irregular obstacles. This paper presents a continuous bidirectional Quick-RRT* (CBQ-RRT*) algorithm based on the bidirectional RRT (Bi-RRT) and Quick-RRT* algorithms and proposes an expansion cost function that evaluates path smoothness and length to overcome the limitations of the Quick-RRT* algorithm for non-holonomic mobile robot applications. To improve the zigzag between dual trees caused by the dual-tree expansion of the Bi-RRT algorithm, CBQ-RRT* proposes the CreateConnectNode optimization method, which effectively solves the path smoothness problem at the junction of dual trees. Simulations conducted on the ROS platform showed that the CBQ-RRT* outperformed the unidirectional Quick-RRT* in terms of efficiency for various orchard layouts and terrain conditions. Compared to Bi-RRT*, CBQ-RRT* reduced the average path length and maximum heading angle by 8.5% and 21.7%, respectively. In addition, field tests confirmed the superior performance of the CBQ-RRT*, as evidenced by an average maximum path lateral error of 0.334 m, a significant improvement over Bi-RRT* and Quick-RRT*. These improvements demonstrate the effectiveness of the CBQ-RRT* in complex orchard environments.

## 1 Introduction

Mobile robots are garnering increasing attention among researchers as intelligent devices. Owing to their high degree of automation, mobile robots can improve productivity and provide various conveniences. Mobile robots are currently being utilized in various scenarios (Ferguson and Stentz, 2006; Zhao et al., 2014; Ljungqvist et al., 2019; Reid et al., 2020; Strader et al., 2020; Buchanan et al., 2021; Ye et al., 2023). As agricultural automation research has gained widespread attention in recent years, mobile robots have begun to make their way into agricultural settings (Xiong et al., 2017; Blok et al., 2019; Ren et al., 2020). Khajepour et al. (2020) proposed a particle filter algorithm using a laser beam model and Kalman filter algorithm based on line detection for the positioning and navigation of mobile robots in orchards. Qiu and Li (2022) implemented a path-tracking control for orchard mobile robots using a PID algorithm. Yang et al. (2022) used a dataset of orchard road data to train semantic segmentation networks to extract navigation paths and achieve path tracking for orchard robots. However, the studies above focused on orchard robots operating in structured environments with more prominent road features, with less consideration for path planning and obstacle avoidance. In unstructured orchard scenes, it is difficult for mobile robots to make decisions by obvious features or scene markers to realize specific navigation movements. The mobile robot in the orchard needs to perceive the unstructured orchard environment and plan the movement path for the mobile robot in the navigation operation stably and efficiently through the constructed environment map or by obtaining the real-time perception feedback. Therefore, in the field of mobile robot navigation in orchards, path planning is a fundamental and crucial aspect. Path planning refers to the process by which a mobile robot identifies, within the configuration space, an obstacle-free path from its initial configuration to its target configuration. The path must simultaneously satisfy environmental constraints and the kinematic constraints of the mobile robot itself (Siciliano et al., 2008).

In recent years, a substantial amount of research has been dedicated to the path planning conundrum for orchard robots. Jiang et al. (2022) used 3D and 2D LiDAR to map greenhouse orchards and used the Dijkstra algorithm to plan global paths for mobile robots. Tian et al. (2023) proposed an ant colony optimization algorithm to solve multitarget waypoint planning for unmanned aerial vehicles in orchards. Zhang et al. (2023) introduced an improved artificial potential field algorithm for weed-removal robot path planning, improving the safety of robot automation operations. Zhang et al. (2022) conducted LiDAR scans of greenhouse maps and employed the probabilistic roadmap technique to calculate the paths between all target points, consequently establishing comprehensive global paths for navigating multiple operational points. In addition to general-purpose path planning algorithms, researchers have extensively researched path planning algorithms for special orchard target guidance based on the perception of the orchard scene. Liu et al. (2023) proposed a real-time orchard mapping method based on visual odometry-centered position estimation fused with a LiDAR camera and achieved real-time apple global localization using ORB-SLAM3, YOLO-V5, Euclidean clustering filtering, and the local point cloud sliding window method. Xiong et al. (2023) combined the VINS-RGBD SLAM framework with the semantic segmentation algorithm BiSeNetV1 to propose a real-time localization and semantic map reconstruction method for unstructured citrus orchards. Chen et al. (2021) presented a global map-construction method tailored to the nature of orchard-picking tasks using the ORB-SLAM3 algorithm. Chen et al. (2024) proposed a set of vision algorithms for moving target estimation, real-time self-localization, and dynamic harvesting. In addition, a reliable coordination mechanism was established for continuous motion and harvesting behavior.

In large and complex orchard environments, efficiently determining the navigation paths for mobile robots is of concern (Ye et al., 2023). The rapid-exploration random tree (RRT) algorithm is a framework for path planning algorithms widely used by mobile robots. This algorithm iteratively generates feasible paths by incorporating points obtained from random sampling of the state space, providing probabilistic completeness (LaValle et al., 2001). The efficiency of RRT algorithms for path planning has been highlighted in recent studies. Huan et al. (2023) employed the RRT*-connect algorithm to plan the navigation path of a microrobot in a plant–vein environment. Combined with the characteristics of the Dijkstra algorithm for path optimization, Wencheng et al. (2020) improved the RRT algorithm to solve the path planning problem of orchard spraying robots. Castro et al. (2023) proposed an online adaptive path planning solution fusing RRT and deep reinforcement learning algorithms. Despite these advantages, the RRT has inherent limitations due to its random sampling nature, including (1) reliance on a global uniform random sampling strategy, which results in slower convergence due to the lack of guided search (Li et al., 2020); (2) path generation from random sampling and iteration, resulting in non-optimality (Liu et al., 2023); and (3) unsuitability of RRT-generated paths for scenarios involving mobile robot tracking motion because it overlooks kinematic constraints (Webb and Van Den Berg, 2013).

Researchers have refined the basic RRT algorithm to accommodate various application environments and overcome limitations. Kuffner and LaValle (2000) proposed Bi-RRT, a bidirectional search tree that initiates two parallel trees from the start and target states to expedite algorithm convergence to improve the convergence speed of the RRT algorithm. Ye et al. (2021) enhanced the Bi-RRT by incorporating a gravitational algorithm with an adaptive step and gravitational coefficient adjustment into the AtBi-RRT algorithm, which they applied to a picking robot, achieving improved search efficiency and the ability to navigate complex obstacle regions. Tahir et al. (2018) proposed a potentially guided intelligent bidirectional RRT* algorithm that significantly improves convergence speed and memory utilization. To advance the optimization of paths generated through iterative random sampling in the RRT algorithm, Karaman and Frazzoli (2011) introduced the RRT* algorithm that incorporates key processes such as ChooseParent and Rewire and proved its asymptotic optimality. As the number of samples increased, the algorithm gradually approached the optimal solution, with the path acquisition cost converging to the optimum. However, the convergence is slow, especially in complex environments. The informed RRT* algorithm was introduced to enhance convergence efficiency (Gammell et al., 2014). This algorithm restricts the sampling to an elliptical region that narrows as the path length decreases after finding a feasible path. This strategy preserves the probabilistic completeness and asymptotic optimality of RRT*, speeds up convergence, and improves solution quality. Furthermore, Nasir et al. (2013) developed RRT*-Smart to further speed up convergence. The algorithm starts from the sampled nodes and seeks direct, unobstructed connections to ancestral nodes, reducing path costs. Similarly, Jeong et al. (2019) proposed Quick-RRT*, which extends the set of parent nodes beyond the immediate neighbors to include their ancestors. This approach also influences the rewiring process, favoring the alignment of new nodes with nearby parent nodes, improving initial solutions, and achieving faster optimal convergence compared to RRT. Li et al. (2020) combined a potentially guided method and Quick-RRT* to propose the PQ-RRT* algorithm, which achieved faster convergence and better initial path quality than the Quick-RRT*. Moreover, researchers have proposed two approaches to apply RRT algorithms to kinematically constrained navigation scenarios. The first is the consideration of kinematic constraints during the RRT path search process. Webb and Van Den Berg (2013) introduced kinodynamic-RRT*, an asymptotically optimal path planning solution for robots with linear dynamics. Moon and Chung (2014) proposed a dual-tree RRT algorithm for path planning for mobile robots with differential wheels. Wang et al. (2021) proposed the KB-RRT* algorithm, incorporating kinematic constraints to prevent unnecessary node expansion and speed up the feasible path identification for differential-wheel mobile robots. The second approach involves post-processing to mitigate increasing computational complexity during sampling. Methods such as spline fitting maintain the planning speed while producing smooth paths. Elbanhawi et al. (2015) proposed a cubic B-spline smoothing algorithm that ensured curvature continuity while satisfying a robot’s incompleteness constraint. Other smoothing methods, such as Bezier curves, have also been widely used to smooth the initial RRT paths, making them suitable for different motion planning scenarios (Liu et al., 2019; Li and Yang, 2020; Gan et al., 2021).

The conventional directional extension algorithm research pays relatively little attention to the smooth connection of paths at the conjunction between two trees; in the RRT sampling algorithm, processing the dynamics constraint during each iteration will occupy a significant amount of computational resources and increase the planning time, especially in complex environments; in path planning research combining RRT algorithm with spline fitting, relatively few studies consider the corner constraints of path points, resulting in large deviations between the actual trajectory and planned path, which can easily trigger accidental collisions in complex environments. Therefore, applying the existing path planning methods for the path planning problem of orchard mobile robots in complex environments is relatively difficult. Based on the limitations of the existing studies, our primary contributions are presented below.

(1) The path length and maximum heading angle of the path are simultaneously adopted as the path cost according to the mobile robot. Hence, the algorithm is optimized for path smoothness and improved in terms of path executability during the extension process.
(2) The path planning speed is improved by adding a directional extension method to the Quick-RRT* algorithm.
(3) To improve the local path smoothness of the bidirectional extension algorithm in conjunction with the two trees, the CreateConnectNode optimization algorithm is proposed to generate the optimal connection point directly in the conjunction region of the two trees.

The remainder of this paper is organized as follows: *Section 2* discusses the specific implementation of the proposed continuous bidirectional Quick-RRT* (CBQ-RRT*) algorithm. Simulation comparisons and field experiments for B-RRT*, Quick-RRT*, and CBQ-RRT* are described in *Section 3*, and the results are compared and analyzed. Finally, *Section 4* presents the conclusions and discusses the study.

## 2 Methods

In developing path planning algorithms for the autonomous navigation of mobile robots in orchards, the primary considerations are the executability and efficiency of the paths planned by the algorithm and the computational efficiency of the algorithm. This study improves the Quick-RRT* algorithm and introduces a CBQ-RRT* algorithm to meet these requirements. The pseudocode for the CBQ-RRT* algorithm is presented in **Algorithm 1**. Initially, an extended cost function containing the kinematic constraints of the mobile robot is established, which is applied to lines 4 and 11 in **Algorithm 1** to constrain the extension direction of the nodes. The optimal parent nodes are further selected for *X_new_* and *X_new2_* in lines 7 and 14 using this cost function. Then, a bidirectional augmentation method is introduced to accelerate path planning. Finally, an optimization strategy is proposed for connecting dual trees to avoid abrupt connections that violate the kinematic constraints (line 19). Further details of the implementation are presented in the following section.

**Algorithm 1:** CBQ-RRT*.

*1 V_a_ * ← {*X_start_ *}; *V_b_ * ← {** *X_goal_ * **}; *E_a_ * ← ∅; *E_b_ * ← ∅; *T_a_ * ← (*V_a_ *, *E_a_ *); *T_b_ * ← (*V_b_ *, *E_b_ *); 2 **for** *i* ← 0 to *N do* 3 *X_rand_ * ← Sample(); 4 *X_nearest_ * ← NearestVertex(*X_rand_ *, *T_a_ *); 5 *X_new_ * ← Extend(*X_nearest_ *, *X_rand_ *); 6 *σ_a_ * ← Steer(*X_nearest_ *, *X_new_ *); 7 *X_parent_Ta_ * ← ChooseParent(*X_new_ *, *Depth*); 8 **if** *X_parent_Ta_ * ≠ ∅ then 9 *T_a_ * ← Rewire(*T_a_ *, *X_new_ *, ** *X_ancestries_Ta_ * **) 10 **end if** 11 *X_nearest_Tb_ * ← NearestVertex (*X_new_ *, *T_b_ *); 12 *X_new2_ * ← Extend(*X_nearest_Tb_ *, *X_Tb_Extend_ *); 13 *σ_b_ * ← Steer(*X_nearest_Tb_ *, *X_new2_ *); 14 *X_parent_Tb_ * ← ChooseParent(*X_new2_ *, *Depth*); 15 **if** *X_parent_ * ≠ Ø then 16 *T_b_ ←*Rewire(*T_b_ *, *X_new2_ *, ** *X _ancestries_Tb_ * **) 17 **end if** 18 **if** distance(*X_new2_ *,*X_new_ *) ≤ *GoalRadiu*; 19 *X_create_conn_ * ← CreateConnectNode(*T_a_ *, *T_b_ *, *X_new_ *); 20 **end if** 21 **end for**

### 2.1 Cost function of smooth expansion

In the application of mobile robots in orchards, the efficacy of the RRT and its extended variants in path planning has been suboptimal. The heading angles of the generated path nodes have large variations in the path planning phase because of the random nature of the algorithm, which challenges the kinematic constraints of the mobile robot. This leads to significant lateral deviation in the navigation of the robot, contributing to navigation failure. This section proposes a novel path cost function based on the analysis of the kinematic constraints of a mobile robot that mitigates this problem.

**Figure 1:**
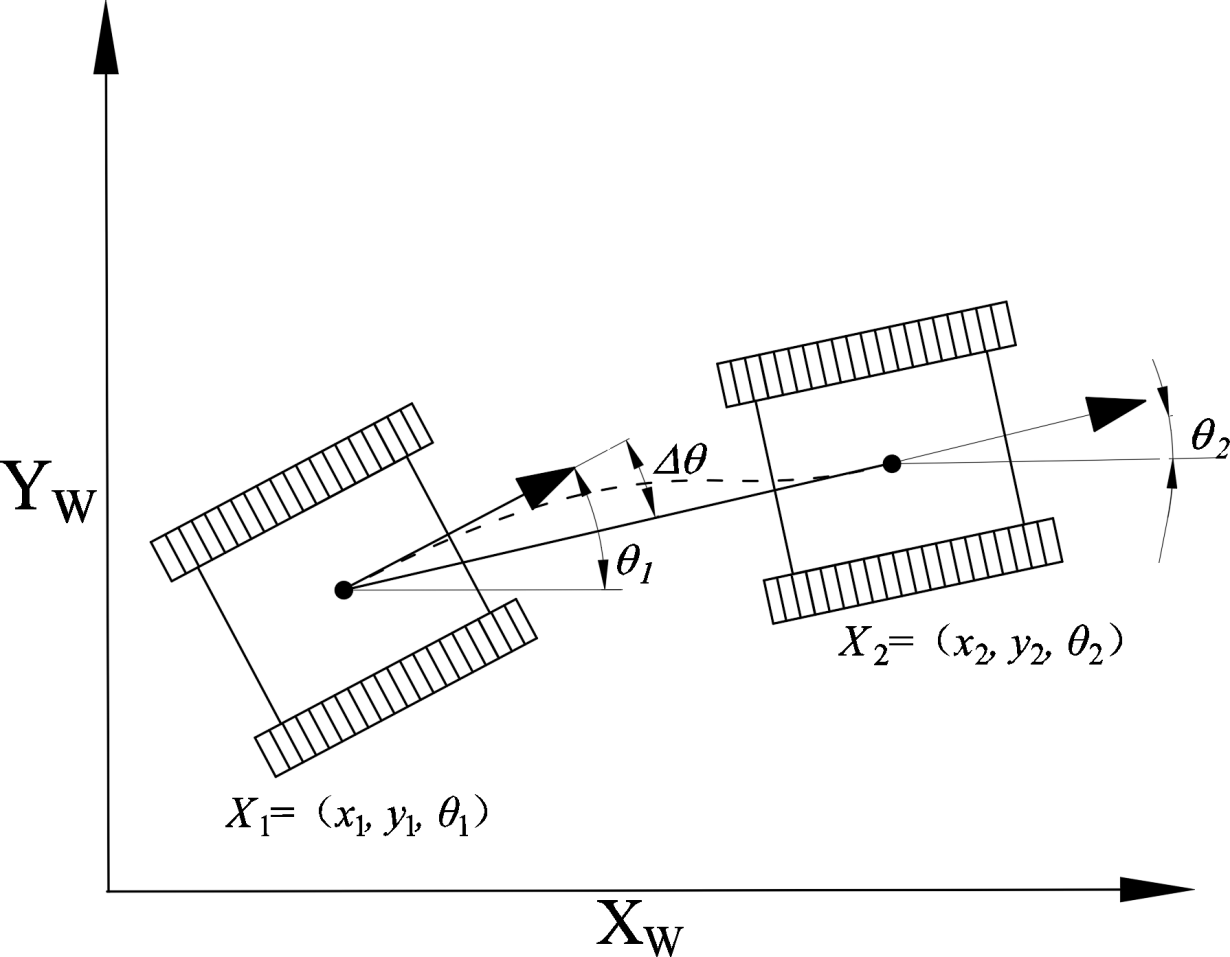
Schematic diagram of the mobile robot configuration.


Assuming a uniform terrain, the spatial position of a tracked mobile robot within an orchard environment can be defined as *X_i_* = (*x_i_*, *y_i_*, *θ_i_*), as illustrated in **Figure 1**. Here, *x_i_* and *y_i_* represent the coordinates in the global *x_w_*–*y_w_* coordinate system, while *θ_i_* signifies the robot’s orientation angle. The computation of *θ_i_* is done using Equation (1).

(1) $$\theta_{i}=\arctan\left(\frac{y_{i}-y_{i-1}}{x_{i}-x_{i-1}}\right)$$

Additionally, by neglecting any track deformation during the motion of the tracked mobile robots, we can approximate their kinematics as that of a differentially driven mobile robot. According to the kinematic model formula, the kinematic constraints can be articulated using Equation (2).


(2) $$\begin{array}{l} \left[\begin{array}{c} x_{i+1}\\ y_{i+1}\\ \theta_{i+1} \end{array}\right]=\left[\begin{array}{c} x_{i}\\ y_{i}\\ \theta_{i} \end{array}\right]+\left[\begin{array}{c} \int_{0}^{\text{Δ}t}v_{c}\cos(\theta_{i}+\omega t)dt\\ \int_{0}^{\text{Δ}t}v_{c}\sin(\theta_{i}+\omega t)dt\\ \int_{0}^{\text{Δ}t}\omega dt \end{array}\right]\\ =\{\begin{array}{c} \left[\begin{array}{c} x_{i}\\ y_{i}\\ \theta_{i} \end{array}\right]+\left[\begin{array}{c} -R\sin\theta_{i}+R\sin(\theta_{i}+\omega \text{Δ}t)\\ R\cos\theta_{i}-R\cos(\theta_{i}+\omega \text{Δ}t)\\ \omega \text{Δ}t \end{array}\right]\omega \neq 0\\ \left[\begin{array}{c} x_{i}\\ y_{i}\\ \theta_{i} \end{array}\right]+\left[\begin{array}{c} v_{c}\text{Δ}t\cos\theta_{i}\\ v_{c}\text{Δ}t\sin\theta_{i}\\ 0 \end{array}\right]w=0 \end{array} \end{array}$$

where *v_c_* and *ω* are the linear and angular velocities of the robot, respectively; *R* is the turning radius of the robot, and $R=\frac{v_{c}}{\omega }$ .

**Figure 2:**
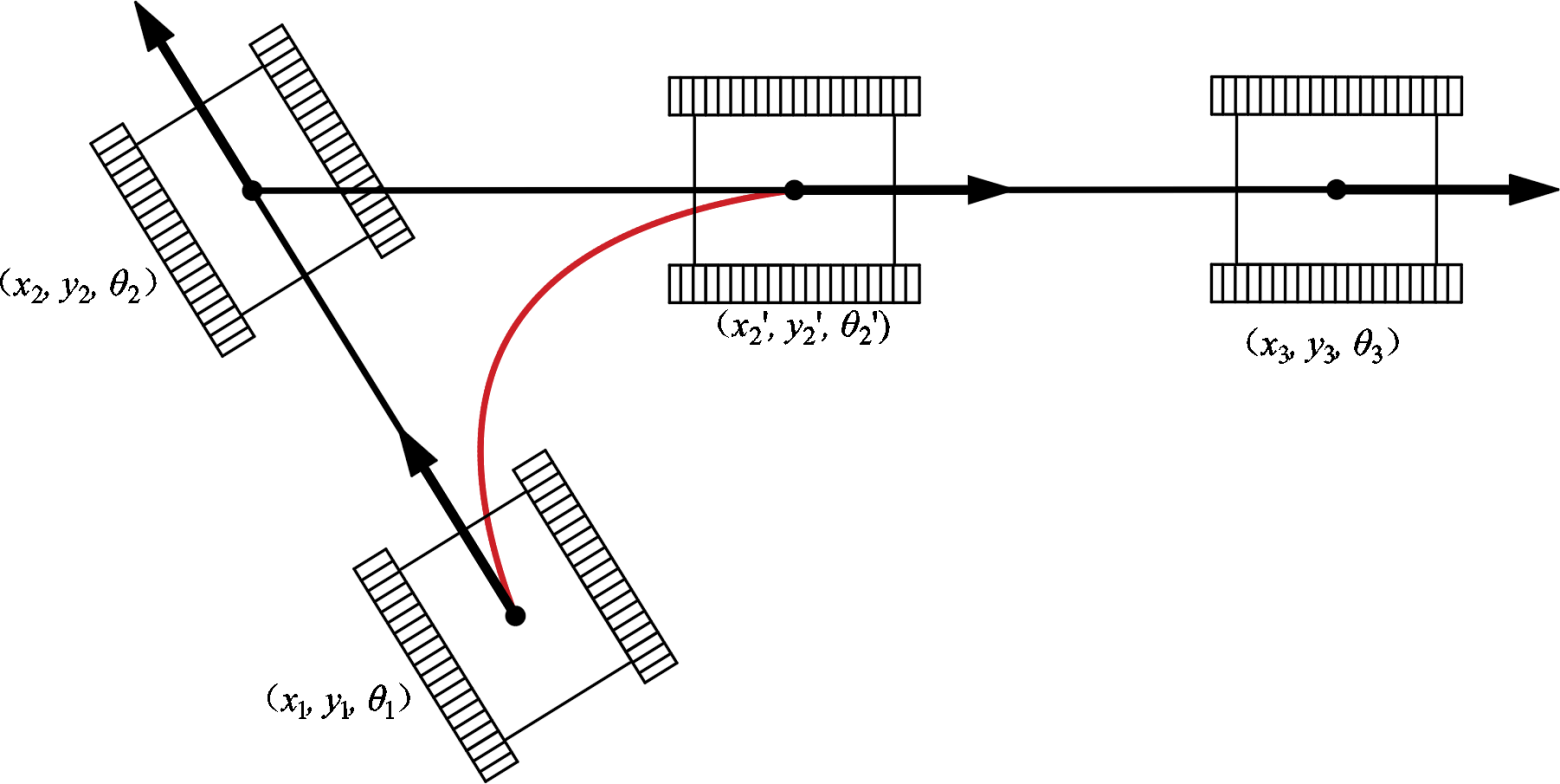
Schematic of mobile robot trajectory deviation.

From Equation (2), the transformation between the robot’s current configurations depends on the kinematic parameter constraints. When the planned path includes points with significant changes in yaw angles, it can result in unreachable points along the path. Consequently, significant lateral deviation errors occur during the robot’s trajectory tracking process, as shown in **Figure 2**, where *X*_2_ = (*x*_2_ *, y*_2_ *, θ*_2_) is the path point of the planned path and *X*_2_’ = (*x*_2_’*, y*_2_’*, θ*_2_’) is the path point reached by the mobile robot under the kinematic constraints.

Thus, significant changes in the heading angle at the waypoint result in considerable lateral error in the path-following trajectory of the mobile robot during navigation. This section introduces a novel path cost function, denoted as *C*(*x_i_*, *x_i+1_*), designed to regulate the expansion direction of the nodes, as shown in Equations (3) and (4).


(3) $$C(X_{i},X_{x+1})=D(x_{i},x_{x+1})+A(X_{i},X_{x+1})$$

(4) $$\{\begin{array}{c} D(X_{i},X_{i+1})={\left(X_{x\_i}-X_{x\_i+1}\right)}^{2}+{\left(X_{y\_i}-X_{y\_i+1}\right)}^{2}\\ A(X_{i},x_{i+1})=\{\begin{array}{c} 0,D(X_{i},X_{i+1})+D(X_{i},X_{i(parent)})<D(X_{i(parent)},X_{i+1})\\ +\infty ,others \end{array} \end{array}$$

where *X_i_*_(_ *_parent_*_)_ denotes the parent node of *X_i_*. The path cost function *C*(*X_i_*, *X_i_*_+1_) comprises a distance cost function *D*(*X_i_*, *x_i_*_+1_) and an angle penalty function *A*(*X_i_*, *X_i_*_+1_). The computation of *D*(*X_i_*, *X_i_*_+1_) involves using the Euclidean distance formula, whereas *A*(*X_i_*, *X_i_*_+1_) is calculated indirectly using the cosine formula. An angle cost penalty is introduced when the heading angle between path nodes exceeds π/2.

**Figure 3:**
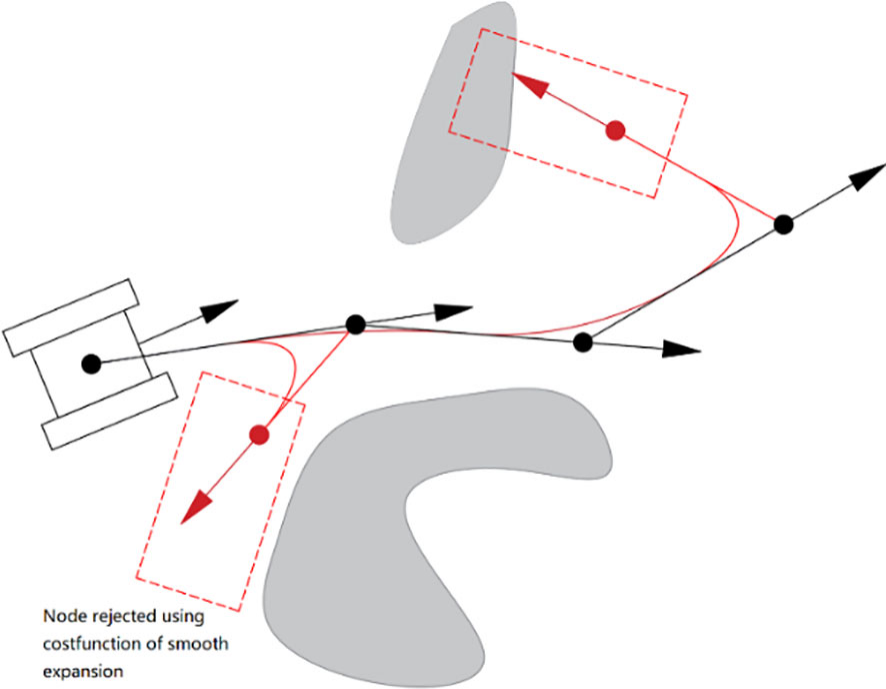
CBQ-RRT* algorithm node expansion process.

The CBQ-RRT* algorithm uses the cost function in lines 4—6 and 11—13 of **Algorithm 1** to select the initial parents of *X_rand_* and *X_new_* in the two extended trees, ensuring that the paths between them and their respective parents satisfy the kinematic constraints. Thus, the CBQ-RRT* algorithm restricts the expansion direction of the nodes, ensuring compliance with the kinematic constraints of the mobile robot. **Figure 3** illustrates the node expansion process. Additionally, the CBQ-RRT* algorithm incorporates the cost function into the ChooseParent and Rewire functions in lines 7–10 and 14–17 of **Algorithm 1**, which further maintains the path to satisfy the kinematic constraints during the path optimization process of the CBQ-RRT* algorithm. The pseudocode for implementing the ChooseParent and Rewire functions is shown in **Algorithm 2** and **Algorithm 3**.

**Algorithm 2:** ChooseParent(*X_new_*, *Depth*).

*1* ** *X_near_ * ** ← NearVertices(*X_new_ *, *T*, *R*); 2 **for** *X_near_ * ∈ ** *X* ** * _near_ * **do** 3 *X_ancestry_ * ← *X_near_ *;4 **for** *i =* 0 to Depth **do** 5 **if** *X_ancestry_ * ∉ ** *X_ancestries_ * **; 6 ** *X_ancestries_ * ** ← *X_ancestry_ * ∪** *X_ancestries_ * **; 7 **end if** 8 *X_ancestry_ * ←parent(*X_ancestry_ *); 9 **end for** 10 **end for** 11 *X_parent_new_ *←findCost_min(** *X_ancestry_ * **, *X_new_ *) 12 **Return** *X_parent_new_ *

**Algorithm 3:** Rewire(***X_new_*** *, X_near_*, X_ancestries_).

**1 for all** *X_from_ *∈*X_new_ *∪** *X_ancestries_ * do** 2 **for all** *X_near_ * ∈** *X_near_ * ** 3 **if** Cost(*X_from_ *) + Cost(*X_from_ *, *X_near_ *) ≤ Cost (** *X* ** * _near_ *) & CollisonFree **then;** 4 *X_parent_near_ * ← *X_from_ *; 5 **end if** 6 **end for**

### 2.2 Dual-tree smooth connected method

**Figure 4:**
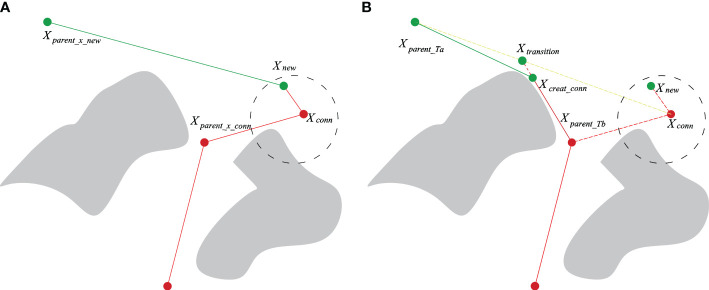
CreateConnectNode algorithm implementation procedure. **(A)** Originally planned path; **(B)** optimized planned path.

Adding the Quick-RRT* algorithm to the bidirectional expansion method in **Algorithm 1** improves the planning efficiency, but in the expansion direction; *T_a_* expands in the *X_rand_* direction and *T_b_* expands in the *X_new_* direction generated by *T_a_*. Moreover, ChooseParent and Rewire optimization during the dual-tree expansion is performed independently, making it difficult to obtain the optimal connection point by random sampling in a complex environment. For example, in **Figure 4**, if the Quick-RRT* algorithm is added directly to the bidirectional expansion method, the iteration is stopped after the dual-tree connection. In addition, there is a higher probability of path points with large changes in the heading angle in the path connection, and a large redundant section of the path is added, as shown in **Figure 4A**. To enhance this situation, once a connection path is obtained between the two trees (line 18 in **Algorithm 1**), the CBQ-RRT* algorithm proposes the CreateConnectNode algorithm to generate a more optimal dual-tree connection point *X_creat_conn_* (line 19 in **Algorithm 1**). The pseudocode implementation is displayed in **Algorithm 4**, and an example of the algorithm implementation is displayed in **Figure 4B**.

**Algorithm 4:** CreateConnectNode(*T_a_*, *T_b_*, *X_new_*).

*1 X_up_ * ← *X_conn_ * 2 *X_bottom_ * ← *X_parent_Ta_ * 3 **while** distance(*X_up_ *, *X_bottom_ *) > *dichotomy* do 4 *X_transition_ * ← (*X_up_ * + *X_bottom_ *)/2; 5 **if** *CollisionFree*(*X_transition_ *, *X_parent_x_new_ *) 6 *X_up_ * ← *X_transition;_ * 7 **else** 8 *X_bottom_ * ← *X_transition_ *; 9 **end if** 10 **end while** 11 *X_bottom_ * ← *X_parent_Tb_ *; 12 **while** distance(*X_up_ *, *X_bottom_ *) > *dichotomy* do 13 *X_creat_conn_ * ← (*X_up_ *+ *X_bottom_ *)/2; 14 if *CollisionFree*(*X_creat_conn_ *, *X_parent_x_new_ *) 15 *X_up_ * ← *X_creat_conn_ *; 16 **else** 17 *X_bottom_ * ← *X_creat_conn_ *; 18 **end if** 19 **end while** 20 **return** *X_creat_conn_ *

**Figure 5:**
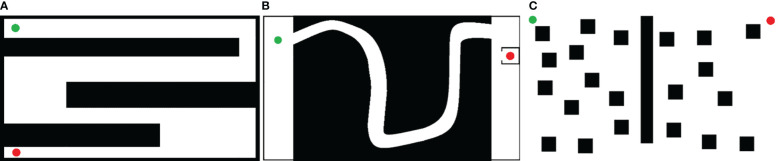
Scenarios of simulation experiment. **(A)** Scenario 1; **(B)** scenario 2; **(C)** scenario 3.

The CreateConnectNode algorithm operates as follows:

(1) When the two expansion trees *T_a_* and *T_b_* are connected, the respective node sets ***X*** *_ancestry_Ta_* and ***X*** *_ancestry_Tb_* belonging to node *X_conn_* are identified. The parent nodes *X_parent_Ta_* and *X_parent_Tb_* of *X_conn_* are also located within this set.
(2) Initially, the transition point *X_transition_* on the line linking *X_parent_Ta_* and *X_conn_* is ascertained. At the iteration onset, *X_parent_Ta_* and *X_conn_* are characterized as the lower and upper endpoints *X_bottom_* and *X_top_*, respectively. The midpoint between *X_bottom_* and *X_top_* is defined as *X_mid_*. This is followed by connecting *X_parent_Tb_* and *X_mid_*. In the absence of a collision, *X_mid_* is reassigned as *X_top_*; otherwise, *X_mid_* is redefined as *X_bottom_*. The iteration continues, and according to the dichotomy principle, the distance between *X_top_* and *X_bottom_* is progressively reduced until it falls below a pre-established dichotomy parameter. Using this approximation, *X_transition_* on the line connecting *X_parent_Ta_* and *X_conn_* is obtained. Applying the same principle, *X_transition_* and *X_parent_Tb_* are designated as *X_up_* and *X_bottom_*, respectively, with the optimal connection point *X_create_conn_* discovered on the line connecting *X_transition_* and *X_parent_Tb_*.
(3) Eventually, *X_create_conn_* serves as the link connecting the two expansion trees Ta and Tb, with *X_parent_Ta_* and *X_parent_Tb_* assigned as parent nodes, respectively, thereby combining the two trees and revealing the final path, as depicted in **Figure 5B**. The CreateConnectNode optimization algorithm produces an improved connection point, *X_create_conn_*, over the original. This resolves the smoothing issue encountered when connecting two expansion trees, thereby reducing both the trajectory’s extensive angular change and its length.

## 3 Simulations and experimentations

### 3.1 Path planning simulations

#### 3.1.1 Simulation setting

**Figure 6:**
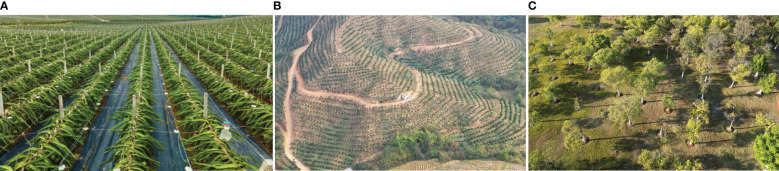
Simulated orchard scene. **(A)** Standardized orchard planting; **(B)** orchard with irregular roads; **(C)** orchard with irregular planting.

To evaluate the performance of the proposed algorithm, three representative orchard scenarios (**Figure 6**) were used to construct the corresponding simulation maps (**Figure 5**). The white and black areas indicate the feasible and obstacle areas, respectively; the green and red points indicate the starting and target points of the scenario path plan, respectively. The maps have dimensions of 1,152×648 pixels and a resolution of 0.025 m/pixel. The starting and target configurations for all three scenarios are presented in **Table 1**. The simulation experiments were conducted on a computer powered by an Intel Core i5 processor with 16 GB of RAM utilizing the ROS-Melodic platform. For a comparative analysis, the proposed algorithm was pitted against the Bi-RRT* and Quick-RRT* algorithms, run with the depth parameter set to three during the simulation.

**Table 1:** Parameters of the starting and target points of the simulation environment.

Scenarios	Starting configuration	Target configuration
1	[0 m, 0 m, 0°]	[0 m,-14 m, 180°]
2	[0 m, 0 m, 0°]	[3 m,27 m, 90°]
3	[0 m, 0 m, 0°]	[26.5 m,-1.6 m, 0°]

In the practical application of the orchard mobile robot navigation path planning, we focused on the executability, execution efficiency of the planned paths, and computational efficiency of the algorithm. Therefore, we systematically recorded the key parameters: the length and maximum steering angle of the algorithmically planned path, and the time taken by the algorithm for path formulation in the simulation. Path length serves as a central metric for evaluating the execution efficiency of an algorithmically planned path. A shorter path length directly corresponds to reduced time to execute the path, particularly for a given linear velocity of the mobile robot. Simultaneously, the maximum steering angle acts as an indicator of path smoothness. The conclusions drawn from the previous kinematic analysis indicate that a smaller maximum steering angle in the path correlates with a smaller lateral error generated by the mobile robot during path execution. The execution time of an algorithm is a direct indicator of its computational efficiency, which is related to its real-time responsiveness to dynamic scenarios.

In this section, each algorithm was executed 100 times in the three scenarios. The evaluation aimed to assess the efficiency of path planning, the feasibility of mobile robots in orchards, and overall algorithmic efficiency.

#### 3.1.2 Analysis of simulation results

**Figure 7:**
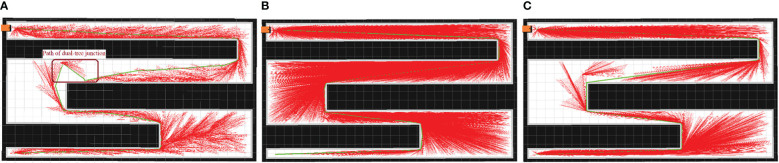
Example of simulation results for scenario 1. **(A)** Bi-RRT*; **(B)** Quick-RRT*; **(C)** CBQ-RRT*.

**Figure 8:**
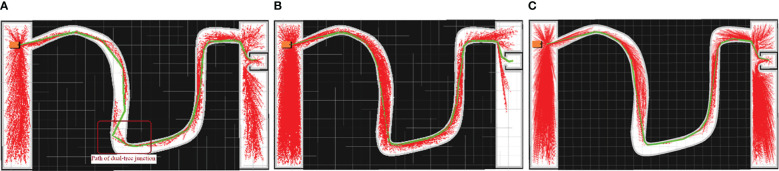
Example of simulation results for scenario 2. **(A)** Bi-RRT*; **(B)** Quick-RRT*; **(C)** CBQ-RRT*.

**Figure 9:**
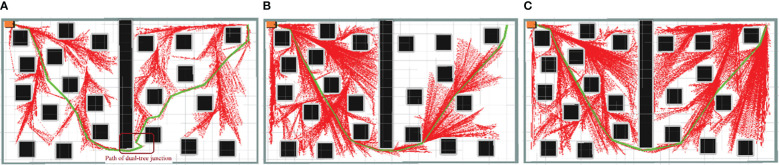
Example of simulation results for scenario 3. **(A)** Bi-RRT*; **(B)** Quick-RRT*; **(C)** CBQ-RRT*.

Examples of the simulation results are shown in **Figures 7**–**9**. The thin red lines indicate the randomly constructed trees, whereas the thick green lines indicate the final planned paths.

**Figure 10:**
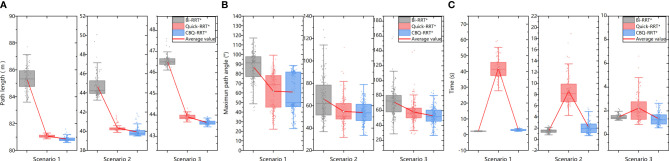
Statistics of simulation results. **(A)** The path length statistics results; **(B)** the maximum path angle statistics results; **(C)** the planning time statistics results.

The path planning results highlight a notable reduction in the number of red lines for the Bi-RRT and CBQ-RRT* algorithms compared to the Quick-RRT* algorithm. This reduction implies that the Bi-RRT* and CBQ-RRT* algorithms can formulate planned paths with an expansion of a relatively modest number of nodes, indicating superior efficiency in path planning. Additionally, an examination of the final planned path, represented by the thick green line, reveals distinctive characteristics. The path of the Bi-RRT* algorithm at the dual-tree node, as indicated by the rectangular box, displays a noticeable curvature. In contrast, the path planned by the CBQ-RRT* algorithm demonstrates remarkable smoothness, with a shorter length than that planned by the Bi-RRT* algorithm. This observation underscores the superior quality of the paths generated by the CBQ-RRT* algorithm compared to the Bi-RRT* algorithm. The simulation examples provided initially demonstrate that the CBQ-RRT* algorithm can achieve higher-quality path planning while maintaining superior algorithmic efficiency. More detailed simulation results and statistics are presented in **Figure 10** and **Tables 2**–**4**.

**Table 2:** Path length statistics of simulation results.

Scenarios Algorithm	Scenario 1	Scenario 2	Scenario 3
CBQ-RRT*	80.84 m	39.93 m	43.65 m
Quick-RRT*	81.06 m (+0.2%)	40.33 m (+1.0%)	43.92 m (+0.6%)
Bi-RRT*	85.33 m (+5.3%)	44.71 m (+10.9%)	46.55 m (+6.0%)

**Table 3:** Maximum path angle of simulation results.

Scenarios Algorithm	Scenario 1	Scenario 2	Scenario 3
CBQ-RRT*	60.89°	53.93°	52.89°
Quick-RRT*	61.74° (+1.4%)	54.78° (+1.6%)	58.26° (+1.0%)
Bi-RRT*	74.33° (+41.44%)	60.46° (+20.4%)	71.61° (+22.9%)

**Table 4:** Planning time statistics of simulation results.

Scenarios Algorithm	Scenario 1	Scenario 2	Scenario 3
CBQ-RRT*	2.96 s	2.14 s	1.33 s
Quick-RRT*	41.97 s	8.86 s	2.24 s
Bi-RRT*	2.15 s	1.47 s	1.49 s

First, **Figure 10A** and **Table 2** demonstrate that the CBQ-RRT* algorithm consistently outperforms the Bi-RRT* and Quick-RRT* algorithms in generating more efficient paths across the three distinct environmental scenarios. During iteration, the CBQ-RRT* and Quick-RRT* algorithms enhance the potential parent node set in the ChooseParent and Rewire phases, finding shorter paths. Additionally, the CBQ-RRT* algorithm employs CreateConnectNode optimization, significantly improving path quality and yielding shorter paths than the Quick-RRT* algorithm, as shown in **Table 2**.

Second, unlike Bi-RRT* and Quick-RRT* algorithms, the CBQ-RRT* algorithm integrates a cost function that accounts for cornering constraints, significantly reducing sudden changes in path curvature. The CBQ-RRT* algorithm uses a smoothing connection technique in the bidirectional tree connection process to produce smoother paths and is further supported by the maximum path-turning angle data in **Figure 10B** and **Table 3**. Based on the constraints imposed by the robot kinematics, the smaller the maximum turning angle, the smaller the lateral deviation error of the mobile robot when executing the path.

Finally, regarding algorithmic efficiency, **Figure 10C** and **Table 4** show that Bi-RRT* and CBQ-RRT* algorithms have shorter path initialization times than the Quick-RRT* algorithm, particularly in Scenario 1, as characterized by the smaller feasible region and a more complex environment. This emphasizes the effectiveness of bidirectional RRT algorithms, including CBQ-RRT*, in meeting the efficiency needs of path planning for mobile robots in orchard settings.

The results confirm that the CBQ-RRT* algorithm is superior to Bi-RRT* and Quick-RRT* algorithms in terms of overall performance and demonstrates its ability to efficiently plan shorter collision-free paths that align better with the kinematic constraints of mobile robots in a shorter time.

### 3.2 Planned path tracking experiment

#### 3.2.1 Experiment setting

**Figure 11:**
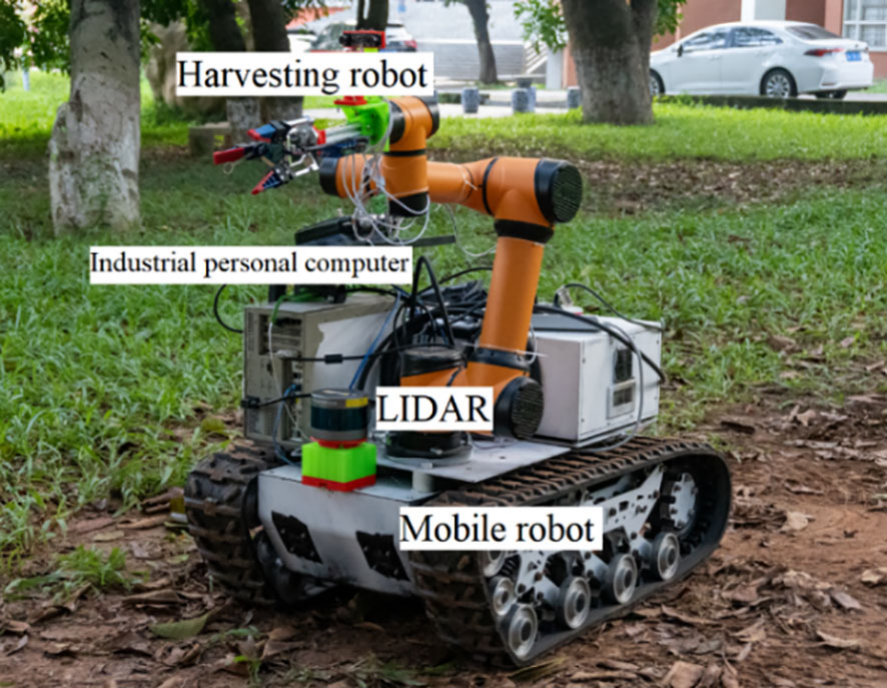
Self-developed mobile robot.

**Figure 12:**
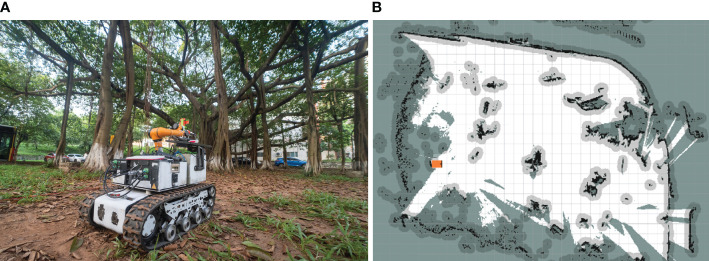
Experimental environment. **(A)** Experimental field scenario; **(B)** grid map of the experimental scenario.

The algorithmic performance of CBQ-RRT* was further evaluated in an orchard context using real-world experiments. The physical orchard mobile robot used is shown in **Figure 11**, and its motion parameters are listed in **Table 5**. The experimental environment consisted of an orchard with complex obstacles, as shown in **Figure 12A**. The mobile robot, equipped for picking, incorporated a three-dimensional LiDAR “Robosensen_16” for environmental sensing and a nine-axis IMU module to monitor its movement. From the resulting sensor data, an offline map is constructed using the cartographer algorithm proposed by Hess et al. (2016) to facilitate path planning. This map, shown in **Figure 12B**, consists of 712×659 pixels at a resolution of 0.05 m/pixel.

**Table 5:** The motion parameters of the mobile robot.

Parameter	Value
Minimum turning radius *R_min_*	0.6 m
Maximum curvature *k_max_*	1.6 m^-1^
Maximum angular velocity *ω_max_*	20°/s
Maximum speed *v_c_max_*	2 m/s

**Figure 13:**
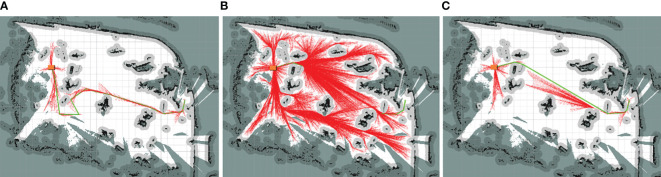
Planned paths result. **(A)** Bi-RRT*; **(B)** Quick-RRT*; **(C)** CBQ-RRT*.

**Figure 14:**
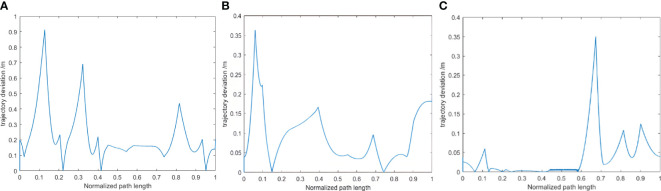
The lateral error records. **(A)** Bi-RRT*; **(B)** Quick-RRT*; **(C)** CBQ-RRT*.

This map was utilized to plan paths using the Bi-RRT*, Quick-RRT*, and CBQ-RRT* algorithms, each originating at [6 m, 2 m, 0°] and terminating at [22.73 m, 1 m, 90°], over five trials per algorithm. The experiments assessed the executability of each path planning algorithm by comparing the lateral deviation error between the planned and actual robot trajectory. The results of these on-field experiments are represented in **Figures 13**, **14**; **Supplementary Figure 1** statistics regarding the maximum path deviation are summarized in **Table 6**.

**Table 6:** Maximum lateral error statistics of the path.

Sequence	Bi-RRT*	Quik-RRT*	CBQ-RRT*
1	0.77 m	0.60 m	0.32 m
2	0.52 m	0.40 m	0.36 m
3	0.92 m	0.37 m	0.42 m
4	1.05 m	0.46 m	0.31 m
5	0.72 m	0.45 m	0.26 m

#### 3.2.2 Analysis of experiment results

**Figure 13** shows the results of the paths planned by the Bi-RRT*, Quick-RRT*, and CBQ-RRT* algorithms in a real environment map. These results are consistent with those obtained in the simulation experiments, with the CBQ-RRT* algorithm demonstrating the ability to generate planned paths of relatively high quality using fewer extended nodes.

The real-time trajectory of the robot was monitored during path execution using the localization module of the Cartographer algorithm. **Supplementary Figure 1** shows the paths planned by the three algorithms, represented by red line segments, along with the actual trajectories of the mobile robot during navigation, represented by blue curves. The planned and actual trajectories are length normalized to quantify the executability of the path during the navigation process. We select *n* observation points from them in equal proportions and calculate the lateral error of the mobile robot during the path-following process as Equation (5).


(5) $$Lateral\_err(i)=\sqrt{{\left[x{\left(i\right)}_{p}-x{\left(i\right)}_{r}\right]}^{2}+{\left[y{\left(i\right)}_{p}-y{\left(i\right)}_{r}\right]}^{2}}$$

where $x{\left(i\right)}_{p}$ , $y{\left(i\right)}_{p}$ and $x{\left(i\right)}_{r}$ , $y{\left(i\right)}_{r}$ are the coordinates of the *i*th observation point in the planned path and actual trajectory, respectively. **Figure 14** shows the lateral error between the actual and planned trajectories when the mobile robot executes the paths planned by these algorithms. **Supplementary Figure 2** illustrates the movement of a mobile robot when executing a planned path. The mobile robot exhibits comparatively smaller lateral errors in **Figure 14**; **Supplementary Figures 1, 2** when executing the paths planned by the CBQ-RRT* algorithm, suggesting superior executability of the paths planned by the CBQ-RRT* algorithm.

**Table 6** presents the maximum path lateral error statistics obtained from these experiments. CBQ-RRT* recorded an average maximum path lateral error of 0.334 m across five trials. In contrast, Quick-RRT* and Bi-RRT* recorded lateral errors of 0.455 and 0.796 m, respectively. The field experiments validated the superior performance of the CBQ-RRT* algorithm in challenging orchards.

## 4 Conclusions

When operating autonomously, orchard mobile robots, such as those used for weeding, spraying, and picking, must easily traverse through the orchard. Existing research on the navigation scheme of orchard mobile robots focuses on path extraction and fixed path following in a structured orchard. However, this type of research cannot be applied to unstructured orchard scenes where the road characteristics are fuzzy, and the robot needs to autonomously plan the navigation path according to environmental perception. Therefore, an efficient path planning algorithm is key to achieving improved automation for mobile orchard robots in unstructured orchard environments.

In this study, based on the Quick-RRT* algorithm, a bidirectional expansion method was introduced, and a computationally lightweight path cost function was proposed to maintain the smoothness of the paths between nodes and the efficiency of node expansion during random tree expansion. Moreover, the algorithm performs smoothness optimization during dual-tree link processing, which locally improves the kinematics of the paths at the dual-tree link. We prove the effectiveness of the CBQ-RRT* algorithm for the path planning problem of working mobile robots in an orchard scene by setting three types of orchard simulation maps: a standardized planting scene, an irregular road scene, and an irregular planting scene. The statistical simulation results of the experiment show that, compared with the Bi-RRT* algorithm, the CBQ-RRT* algorithm improves the length and smoothness of the planned paths under the premise of using a similar path planning time, and the planning efficiency is significantly improved compared with the Quick-RRT* algorithm. Path-following experiments were conducted in a real field environment to prove further the effectiveness of the CBQ-RRT* algorithm in practical work-robot applications. The experimental results show that the mobile robot can follow the path planned by the CBQ-RRT* algorithm more accurately. The experimental results show that the CBQ-RRT* algorithm can effectively plan a safe and feasible point-to-point navigation path for a mobile robot in a complex orchard environment and the mobile robot can follow the path planned by the CBQ-RRT* algorithm more accurately. Autonomous point-to-point navigation is integral in orchard robotics, particularly spraying, weeding, and inspection robots. These robots also share structural similarities with the tracked chassis used in our experiment, leading us to believe that CBQ-RRT* can be directly applied to enhance operational efficiency.

## Data availability statement

The original contributions presented in the study are included in the article/**Supplementary Material**. Further inquiries can be directed to the corresponding author.

## Author contributions

LY: Conceptualization, Methodology, Software, Writing – original draft. JL: Resources, Writing – review & editing. PL: Data curation, Writing – review & editing.
