# Supplementary material for: Improving path planning for mobile robots in complex orchard environments: the continuous bidirectional Quick-RRT* algorithm
Source: Front Plant Sci. 2024 May 13;15:1337638. doi: 10.3389/fpls.2024.1337638 (PMC11128624; doi:10.3389/fpls.2024.1337638)
Supplement: Supplementary file 1 [file DataSheet_1.docx]

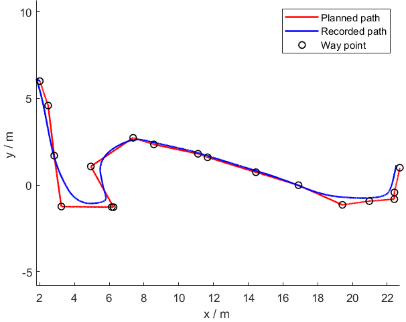

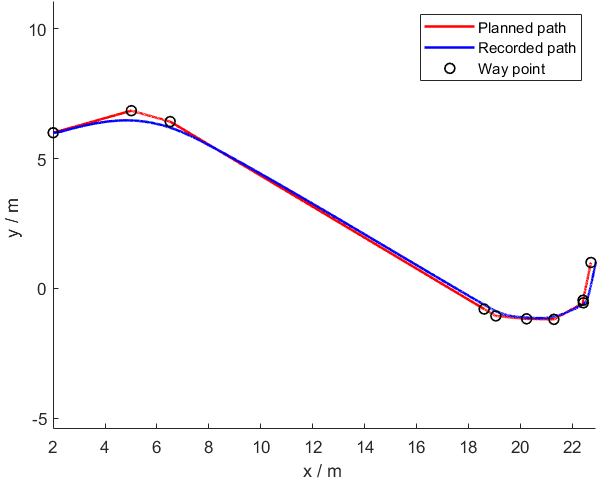

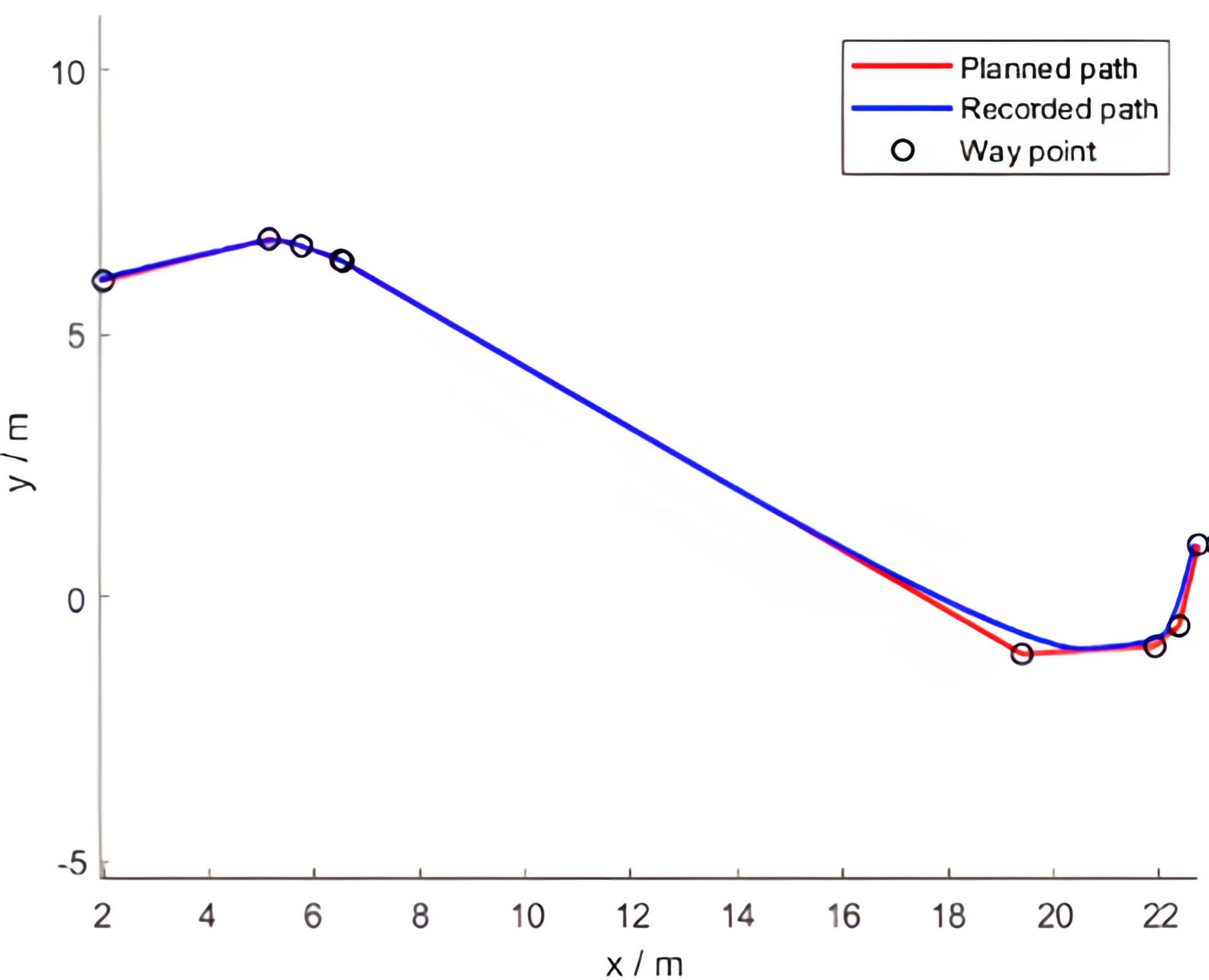


(a) Bi-RRT (b) Quick-RRT (c) CBQ-RRT

**Supplementary Figure 1** Trajectory records.


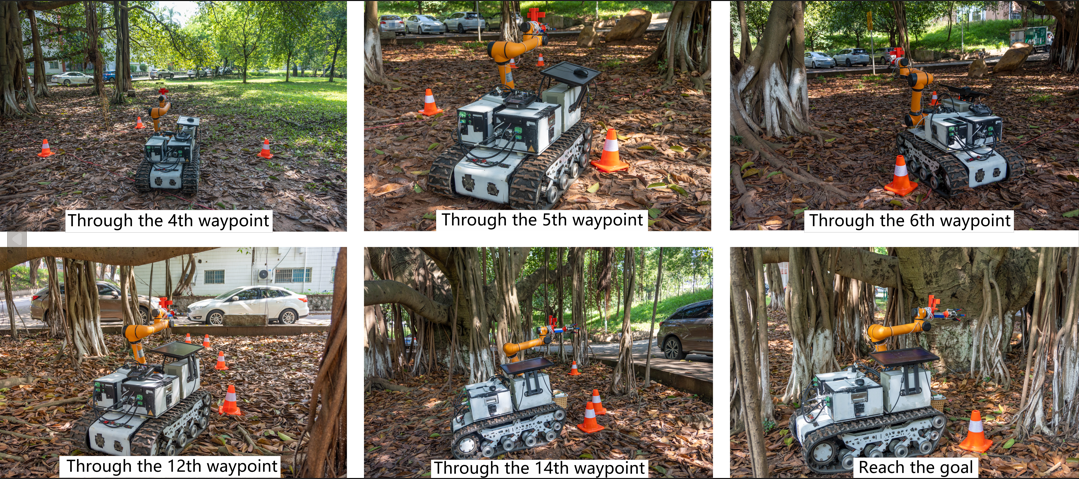


(a) Bi-RRT*


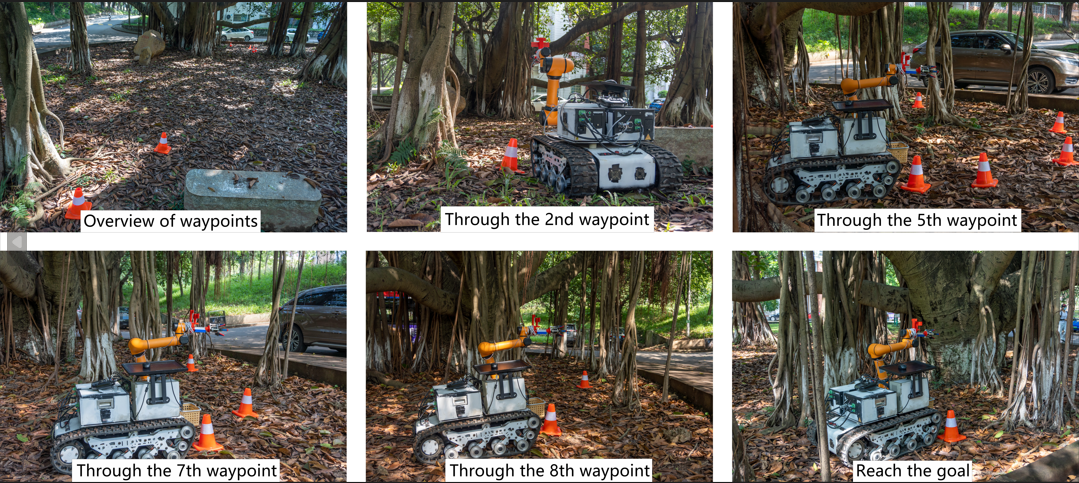


(b) Quick-RRT*


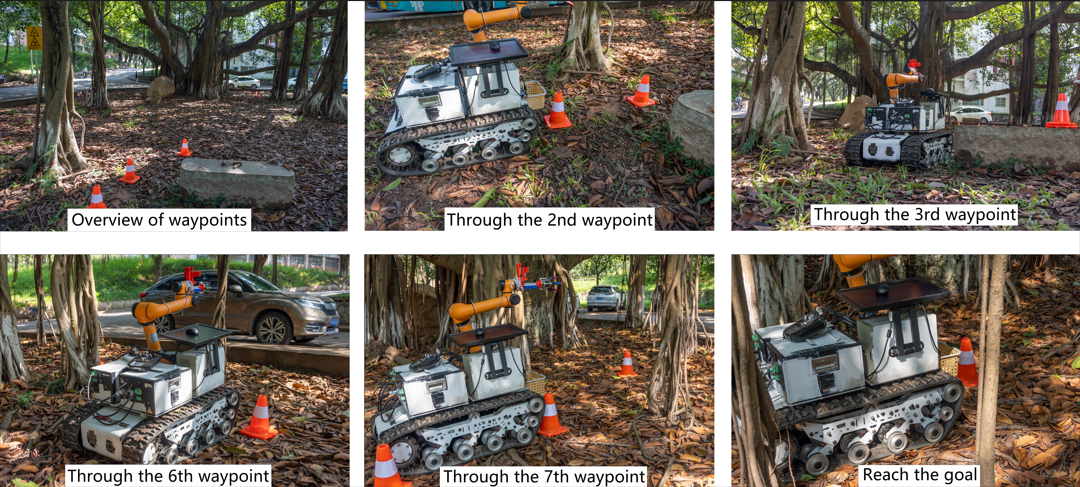


(c) CBQ-RRT*

**Supplementary Figure 2** Path implementation process.
